# Forward layer-wise learning of convolutional neural networks through separation index maximizing

**DOI:** 10.1038/s41598-024-59176-3

**Published:** 2024-04-13

**Authors:** Ali Karimi, Ahmad Kalhor, Melika Sadeghi Tabrizi

**Affiliations:** School of Electrical and Computer Engineering, College of Engineering, University of Tehran, Tehran, Iran

**Keywords:** Computational science, Computer science

## Abstract

This paper proposes a forward layer-wise learning algorithm for CNNs in classification problems. The algorithm utilizes the Separation Index (SI) as a supervised complexity measure to evaluate and train each layer in a forward manner. The proposed method explains that gradually increasing the SI through layers reduces the input data’s uncertainties and disturbances, achieving a better feature space representation. Hence, by approximating the SI with a variant of local triplet loss at each layer, a gradient-based learning algorithm is suggested to maximize it. Inspired by the NGRAD (Neural Gradient Representation by Activity Differences) hypothesis, the proposed algorithm operates in a forward manner without explicit error information from the last layer. The algorithm’s performance is evaluated on image classification tasks using VGG16, VGG19, AlexNet, and LeNet architectures with CIFAR-10, CIFAR-100, Raabin-WBC, and Fashion-MNIST datasets. Additionally, the experiments are applied to text classification tasks using the DBPedia and AG’s News datasets. The results demonstrate that the proposed layer-wise learning algorithm outperforms state-of-the-art methods in accuracy and time complexity.

## Introduction

There has been a significant advancement in machine learning over the past few years, and deep neural networks based on backpropagation learning^1–3^ have gained prominence in many problems. Many different domains have been able to benefit from their use, including image and speech recognition^4–6^, text processing^7^, activity recognition^8^, language translation^9^, medical issues specially Covid19^10,11^. As a fundamental learning algorithm, End-to-End learning is widely used in supervised learning. However, its applications extend beyond supervised learning to include unsupervised^12^, self-supervised^13^, and semi-supervised^14^ methods. While there is currently no direct evidence supporting the utilization of a backpropagation-like algorithm in the brain for learning, previous studies have demonstrated that models trained with backpropagation can effectively explain observed neural responses. For instance, the response properties of neurons in the posterior parietal cortex^15^ and primary motor cortex^16^ align well with the predictions made by backpropagation-trained models.

The fundamental idea is that the brain achieves effective synaptic updates by utilizing feedback connections to induce neuron activities. These activities, computed locally, encode error signals similar to backpropagation. The hypothesis of NGRAD^17^ is based on the idea that higher-level activities, originating from sources such as a target, another modality, or a broader spatial or temporal context, can drive lower-level activities towards values that align with the higher-level activity or desired output. The resulting changes in lower-level activities can then be used to compute weight updates similar to backpropagation, relying solely on locally available signals. Thus, the fundamental notion is that top-down activities drive learning, obviating the need for explicit error information propagation between layers^18^.

The SI^19^ is a distance-based metric for evaluating input data and its transformation across layers in a convolutional neural network tailored for classification tasks. Distinguishing itself from traditional shape-based indices, SI is shape-less and grounded in core principles of class separability and data complexity inherent to classification challenges. Data complexity manifests when a dataset contains samples residing in overlapping regions across different classes, presenting a classification hurdle. Conversely, a class is considered separable when its samples exhibit distinct positions, avoiding overlap. SI quantifies dataset complexity by tallying samples in the overlapping region, normalized against the total sample count. The SI can be utilized for evaluating datasets^19^, ranking neural network models^20^, and assessing the training progress of neural networks^21^. However, this index has not been directly employed for training networks. We in this paper introduce a loss function based on a triplet loss, where loss function terms are explicitly defined using the SI.

The proposed model introduces a layer-wise learning algorithm inspired by the NGRAD hypothesis^18^, emphasizing a biological perspective. This algorithm enables forward training of all layers without carrying explicit error information from the last layer, reflecting the brain’s mechanism of effective synaptic updates through feedback connections and locally computed activities encoding error signals. The integration of the SI plays a pivotal role in this process, aligning with the NGRAD hypothesis. The SI, acting as a nudge from target labels, contributes to the learning trajectory of each layer, addressing a necessary condition for effective training. Despite not explicitly representing errors between network outputs and targets, the SI minimizes disturbances and data sparsity, providing internal feature representations in line with backpropagation goals. This approach establishes a nuanced connection between artificial neural networks and biological learning processes, underscoring the potential biological relevance of the proposed model.

**Figure 1:**
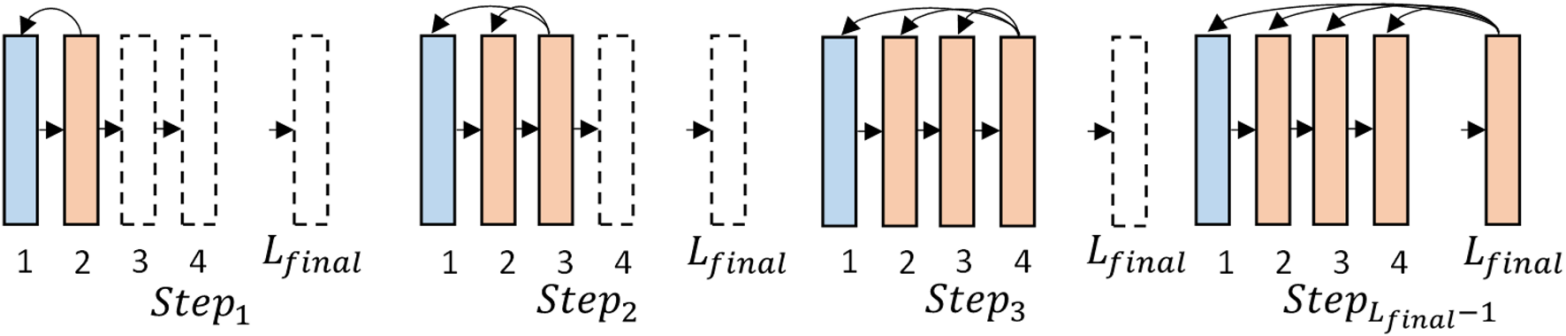
A scheme of layer-wise learning algorithm by SI maximizing.

Since SI has a cardinal-based definition, it is necessary to increase it using gradient algorithm methods. In this paper, the SI is approximated as a variant of a triplet loss, where positive and negative examples of each anchor are its nearest neighbors with the same and different labels, respectively. Consequently, by employing such a triplet loss and applying a local error-backpropagation algorithm, the SI increases layer by layer. Thus, a forward-backward learning algorithm for a CNN is established, where all layers are trained sequentially, as depicted in Fig. 1.

In the following, Section “Related works” provides a comprehensive review of related works in layer-wise learning. In Section “Proposed Method”, we introduce the method. Experimental results and performance evaluation are presented in Section “Experimental results”. Finally, we conclude the paper in Section “Conclusion” by summarizing our contributions and highlighting the significance of our proposed approach.

## Related works

The Forward-Forward algorithm, introduced by Hinton^22^, presents a novel learning procedure for neural networks, deviating from the conventional backpropagation method. Instead, it employs two forward passes: one with positive data and another with negative data. Each layer is equipped with its loss function in this approach, aiming for high goodness with positive data and low goodness with negative data. This innovative algorithm introduces a shift from the traditional backpropagation paradigm, providing a unique perspective on optimizing neural network learning.

Bengio et al.^23^ propose a layerwise algorithm that is supervised and greedy. Each new hidden layer is trained as a hidden layer for a supervised network, with a hidden layer used as an input for the previous layer’s output, and the output of the last layer is discarded. The performance of the MNIST dataset was assessed using five different algorithms in an experiment: (1) DBN (Deep Belief Network), (2) a deep network with layers initialized as auto-encoders, (3) a supervised greedy layer-wise algorithm for pre-training each layer, (4) a deep network with no pre-training, and (5) a shallow network.

During the test, it was observed that the error rate of the training data in the unsupervised greedy pre-trained mode was lower than that of the deep network without pre-training, indicating better performance. Furthermore, a comparison was made between two greedy layer-wise modes, supervised and unsupervised, revealing that the unsupervised technique outperformed the supervised one.

However, this paper, despite introducing a novel learning method, faces a limitation. The lack of training for previous layers during the layer-wise training process results in inadequate learning for these preceding layers. As indicated by the results, this deficiency leads to a decline in the final accuracy of neural networks.

Li et al.^24^ propose a stage-wise learning approach based on unsupervised learning. The stage-wise learning partition method divides the task into three stages: initial, middle, and late partitions. The initial stage tackles simpler tasks, producing features of higher quality in the final stages.

The learning method focuses on each specific target stage rather than requiring the entire network to learn the final target. This approach has several advantages. First, the initial stage is tasked with learning a simpler task. Second, the final stage can leverage the advantages of the initial stage through weight sharing. Third, backward propagation between each stage takes a shorter path compared to the end-to-end learning method. Although this paper has conducted extensive experiments across various topics, showcasing the applicability of the proposed method in different domains, it is important to note that the approach operates in a stage-wise manner rather than a layer-wise one. In each stage, it trains multiple layers instead of a single layer.

The greedy approach^25^ requires less access to the overall gradient, which can have several advantages. One advantage is that there is no need to store intermediate activations and gradients. Sequential learning in shallow networks can serve as an alternative solution for end-to-end learning with backpropagation. With this learning approach, it is possible to observe the progress of each layer in terms of linear separability. Unfortunately, the existing regularization methods do not achieve satisfactory accuracy for large datasets like ImageNet.

The innovation of this paper lies in the use of auxiliary tasks in each layer, where feature reduction takes place through these auxiliary tasks in each layer. This layer-wise learning method has been able to compete with end-to-end learning methods for the AlexNet and VGG networks on CIFAR-10 and ImageNet datasets. The authors of this paper concluded that solving auxiliary problems sequentially with a hidden layer leads to a CNN that performs better than AlexNet on the ImageNet dataset. Then, this idea was expanded, and by solving auxiliary problems with 2 or 3 hidden layers (k-hidden layer CNN problem, k=2,3), an 11-layer model was obtained that outperforms some models from the VGG family. It is claimed that by training a VGG-11 model, the same accuracy as the end-to-end learning method can be achieved. It is claimed that this alternative approach is the first one to compete with the end-to-end learning method on the ImageNet^26^ dataset.

Ko et al.^27^ proposed a method that increases the learning rate more than it should for layers with less variance gradient than the entire model, and conversely, it decreases the learning rate less than it should for layers with a greater learning rate than the entire model. With fewer iterations, this method can achieve better accuracy than other learning rate scaling methods, except for CIFAR-10 with a batch size of 1024. While the proposed method has significantly improved performance on the CIFAR-100 dataset, its results show no substantial improvement on CIFAR-10. In practical terms, its performance has not demonstrated considerable enhancement in datasets with fewer labels.

Yu^28^ et al. presented a different architecture of CNNs for LVSR (Large Vocabulary Speech Recognition). Their method based on layer-wise context expansion and location-based attention. Moreover, like ResNet, there is a connection between some layers. Also, average pooling and max pooling are not used in this method. The results of the experiments show that this model has been able to reduce the error rate compared to DNN and LSTM. However, their experiment is limited to audio and only on few models.

In another research^29^, the authors propose a new approach to train deep neural networks more efficiently and effectively. The proposed method involves training a stacked autoencoder and a stacked-randomized one. This approach entails training the neural network one layer at a time, with each layer learning features subsequently used as input for the next layer. By employing this approach, the neural network can acquire complex representations of protein sequences that prove useful for predicting protein-protein interactions. Their work is valuable from a medical perspective and represents the first study conducted in the field of layer-wise learning in medicine.

Xiong et al.^30^ propose a method for learning the representation in each stage of a network in such a way that the encoder is divided into several modules, each of which has a contrastive loss function at the end. The input is forward-propagated as normal, but the gradients are not back-propagated between modules; instead, each module is greedily trained by a local contrastive loss function. The experiments of this paper have been conducted on the ImageNet dataset, demonstrating the superiority of the results of this method compared to other state-of-the-arts methods.

Tang et al.^31^ presented a method for human activity recognition using wearable sensors and CNNs. The data is preprocessed and segmented into smaller windows fed into the CNNs.The authors then discuss the limitations of traditional CNN architectures for processing wearable sensor data, which typically involves dealing with high-dimensional data with many channels. To address this issue, the authors propose using smaller filter sizes in the convolutional layers, which can capture more detailed information from the input data. The proposed method involves training one layer at a time, starting from the input layer and adding one layer until the entire network is trained. The authors demonstrate the effectiveness of the proposed method on two benchmark datasets and compare it with other state-of-the-art methods. The results show that the proposed method achieves competitive results with significantly fewer training samples, demonstrating its effectiveness for training deep convolutional networks with small datasets.

Horton et al.^32^ propose a novel method for compressing CNNs named ’Layer-Wise Data-Free CNN Compression.’ This method independently compresses each network layer without utilizing any data. Their approach offers several advantages over existing compression methods, particularly its data-free nature, making it more practical and flexible in scenarios where data is scarce or sensitive. The proposed method has significant implications for resource-constrained applications.

Dey et al.^33^ proposed a methodology for making an MLP robust concerning link failures, multiplicative noise, and additive noise by penalizing the system error with three regularizing terms. The approach was tested on ten regression and ten classification tasks with different scenarios of weight alteration, and the experimental results demonstrated the effectiveness of the proposed regularization in achieving robust training of an MLP. The authors also discussed the importance of the coefficient vector in achieving robustness, highlighting its role in different scenarios and its impact on the task and dataset used. Moreover, In^34^ a methodology was presented to improve the robustness of Radial Basis Function Networks (RBFN) against input noises, both additive and multiplicative. This is accomplished by selecting optimal centers and widths for the RBF units in the hidden layer. A combination of Self-Organizing Maps (SOM) is used for center selection, while a nearest-neighbor approach is used for determining the widths. The proposed method aims to make the RBFN less sensitive to input perturbations and outliers, ultimately improving its performance in noisy environments. Experiments were conducted on standard datasets to demonstrate the effectiveness of the approach, showing its superiority over existing methods.

## Proposed method

The proposed method algorithm, presented in algorithm 1, outlines a step-by-step approach to improve the training of neural networks. First, we define the dataset for training and testing and choose a suitable neural network model. Next, we add a new layer to the network. Then, we create pairs of data instances for training, emphasizing the importance of data preparation. The network is trained using a specified loss function. We check for untrained layers and go back to adding layers if needed. After all layers are trained, a classification layer is added and trained using the cross-entropy^35^ loss function. Finally, the algorithm evaluates the accuracy of the trained network on a test dataset. This systematic approach enhances the effectiveness of the proposed method in improving neural network training.

### Separation index in convolutional neural networks

When evaluating the complexity of data in a classification problem, some practical measures have been used in three main categories: measures of overlaps of different classes, measures of the separability of classes, and measures of geometry, topology, and density of classes. Here, SI^19^, as a measure of the separability between classes, is proposed to evaluate and learn layers in a deep neural network. SI quantitatively computes the separation of data points with different labels from the point of view of the nearest neighbor model. For a classification problem with *K* different classes, assume $\textrm{D}=\left\{ \left( {\varvec{x}}_i, l_i\right) \right\} _{i=1}^m$ includes *m* pairs of input patterns $\left( x_i s\right)$ and their ground truth labels $\left( l_i s\right)$ . The SI is defined as follows:

1 $$\begin{aligned} {\text {SI}}(\textrm{D})=\frac{1}{m} \sum _{i=1}^m \delta _K\left( l_i-l_{i^*}\right) \quad i^*=\underset{\forall {\hat{i}} \ne i}{\arg \min }\left\| {\varvec{x}}_i-{\varvec{x}}_{{\hat{i}}}\right\| \end{aligned}$$

where $\delta _K$ operates as Kronecker delta function:

2 $$\begin{aligned} \delta _K(\Delta l)=\left( \begin{array}{cc} 1 &{} \Delta l=0 \\ 0 &{} \text{ else } \end{array}\right. \end{aligned}$$

According to Equation (1), SI counts all data points whose nearest neighbors have the same labels as them. In a classification problem with a real-world dataset, there are some issues, such as different appearances of features, common features between different classes, and various measurement uncertainties and disturbances. Such issues cause examples with the same labels to lose their spatial nearness and become far together, which leads lower SI of the dataset. To increase SI in such a situation, CNNs seem suitable models. CNNs can gradually filter disturbances and common features through their sequenced convolution and pooling layers.

**Figure 2:**
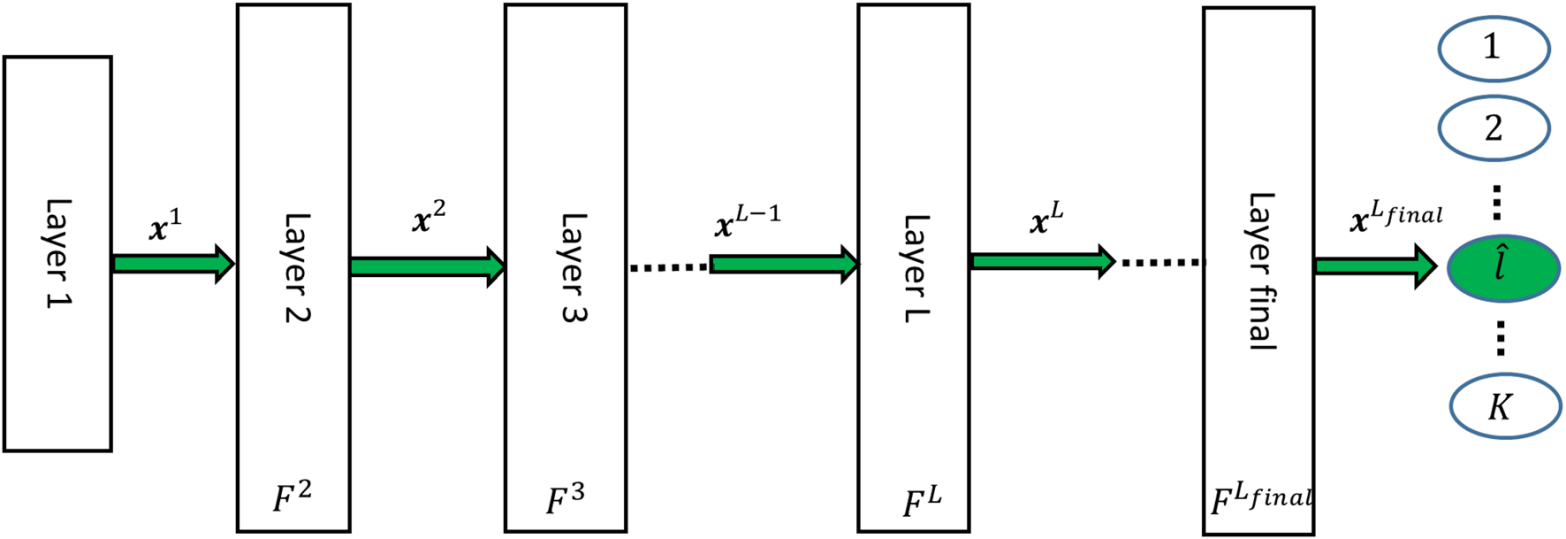
A scheme of convolutional neural networks including the filter part and the nearest neighbor model to predict the labels in classifying different classes.

To formulate and explain such an advantage, consider a CNN with $L_{final}$ layers. By applying the input x to the CNN, it flows through the layers of the model and finally, the predicted label is computed at layer $L_{final}$, as seen in Fig. 2. In this CNN, $x^1$ is equal to *x* and ${\varvec{F}}^L$ denotes the function of layer L on $x^{\textrm{L}-1}$ .

Consider that $\textrm{D}^{\textrm{L}}=\left\{ \left( {\varvec{x}}_i^L, l_i\right) \right\} _{i=1}^m$ denotes the dataflow of D at the $L_{th}$ $\left( \textrm{L} \in \left\{ 1,2, \ldots , \textrm{L}_{\textrm{final}}\right\} \right)$ layer. One can compute SI of $\textrm{D}^{\textrm{L}}$ to measure and evaluate the separability of data points at the $L_{th}$ $\left( \textrm{L} \in \left\{ 1,2, \ldots , \textrm{L}_{\textrm{final}}\right\} \right)$ layer. Now, assume that $i_{th}$ input data point, $x_i^1$, is defined as follows:

3 $$\begin{aligned} {\varvec{x}}_i^1={\varvec{g}}_i\left( \widetilde{{\varvec{x}}}_i{ }^1, {\varvec{p}}_i\right) +{\varvec{d}}_i \end{aligned}$$

Where $\widetilde{{\varvec{x}}}_i^1$ denotes the exclusive features of the $i_th$ input pattern to a certain class, ${\varvec{g}}_i$ denotes the function which defines the appearance form of $\widetilde{{\varvec{x}}}_i^1$, ${\varvec{p}}_i$ denotes the physical measurement parameters of $\widetilde{{\varvec{x}}}_i^1$ and ${\varvec{d}}_i$ denotes the additive effect of disturbances and common features on the ith input. In addition, assume $\overline{{\varvec{F}}}^L(\cdot )$ denotes all cascaded functions from layer one to layer L which operates on $\widetilde{{\varvec{x}}}_i^1$ . Then, we have:

4 $$\begin{aligned} {\varvec{x}}_i^L=\overline{{\varvec{F}}}^L\left( {\varvec{g}}_i\left( \widetilde{{\varvec{x}}}_i{}^1, {\varvec{p}}_i\right) \right) ={\varvec{F}}^L\left( {\varvec{F}}^{L-1} \left( \ldots \left( {\varvec{F}}^2\left( {\varvec{g}}_i \left( \widetilde{{\varvec{x}}}_{i}{}^1, {\varvec{p}}_i\right) \right) \right) \right) \right) \end{aligned}$$

It is feasible that by using a well trained CNN, two conditions are satisfied: (1) the norm of disturbances and common features will decrease layer by layer, and (2) the exclusive features will intensify and scale layer by layer where some data points with the same labels become nearest neighbors:

5 $$\begin{aligned} \text{ If } L \rightarrow L_{final} \text{ then } \overline{{\varvec{F}}}^L\left( {\varvec{g}}_i\left( \tilde{{\varvec{x}}}_i^1, {\varvec{p}}_i\right) +{\varvec{d}}_i\right) \rightarrow \overline{{\varvec{F}}}^L\left( {\varvec{g}}_i\left( \tilde{{\varvec{x}}}_i^1, {\varvec{p}}_i\right) \right) \end{aligned}$$

6 $$\begin{aligned} \forall i: \exists i^* \ne i \text{ where } \text{ If } L \rightarrow L_{final} \text{ then } \left\| \overline{{\varvec{F}}}^L\left( {\varvec{g}}_i \left( \widetilde{{\varvec{x}}}_i^1, {\varvec{p}}_i\right) \right) -\overline{{\varvec{F}}}^L \left( {\varvec{g}}_{i^*}\left( {\widetilde{{\varvec{x}}}_{i^*}}^1, {\varvec{p}}_{i^*}\right) \right) \right\| \rightarrow 0 \end{aligned}$$

By satisfying the given rules in Equation (5) and (6), it is expected that all data points with different labels become far together and all data points with the same labels converge together layer by layer on one or some regions. Regarding the definition of SI in Equation (1), one can say ${\text {SI}}\left( \textrm{D}^{\textrm{L}}\right)$ will increase when $L \rightarrow L_{final}$ .

### A forward layer-wise learning algorithm via triplet loss

Considering the absence of classification layers, it is necessary to utilize loss functions such as triplet loss or similar ones to ensure effective learning and preservation of relative similarities among data points. Triplet Loss is a commonly used metric learning loss function that aims to enhance the feature space by pulling similar instances closer and pushing dissimilar ones apart. It involves forming triplets of data points: an anchor, a positive (similar) sample, and a negative (dissimilar) sample. In various cases such as face verification^36^, vehicle verification^37^, this loss function could perform better from cross-entropy.

The loss function penalizes the model when the distance between the anchor and the positive sample is not smaller than the distance between the anchor and the negative sample by a specified margin. This process encourages the model to learn embeddings that effectively capture the desired similarity relationships in the data. Here, we intend to leverage the capability of Triplet loss for network training, but with modifications to the original formulation. We aim to choose the terms of the formula based on SI for increase SI.

SI is an indicator of the relationship between SI and accuracy. Our objective at each layer is to elevate SI to achieve high accuracy at the end of the network training. Although SI is not gradient-descent-friendly, we have effectively incorporated it into training by defining a variant of triplet loss function. In our approach, the positive sample is the closest data point with the same label, and the negative sample is the closest data point with a different label. We employ the Near-near data selection and we define a new loss based on triplet loss.

Since the proposed SI shows the accuracy of the nearest neighbor model and it does not require any auxiliary classifier, one can develop a forward learning strategy that maximizes the SI at each layer. Regarding to the considered CNN at Fig. 2, assume that the layer $L(L>1)$ is a convolution layer and ${\varvec{x}}^L={\varvec{F}}^L\left( {\varvec{x}}^{L-1}, {\varvec{a}}^L\right)$ where ${\varvec{a}}^L$ denotes all parameters of the convolution operations. To increase SI at such layer L, it is enough to change ${\varvec{a}}^L$ in order that any examples $x_{i^*}$ finds its nearest neighbor $x_{i^*}$ with the same label i.e. $l_i=l_{i^*}$ . Here, a variant of triplet loss without any margin distance is proposed as an alternative to maximizing SI at layer L:

7 $$\begin{aligned} J_{\text{ triplet }}^{\textrm{L}}=\sum _{i=1}^m\left( \left\| {\varvec{x}}_ {{\varvec{i}}}^L-{\varvec{x}}_{{\varvec{i}}_{{\varvec{p}}}}^L\right\| ^2 -\left\| {\varvec{x}}_{{\varvec{i}}}^L-{\varvec{x}}_{{\varvec{i}}_ {{\varvec{n}}}}^L\right\| ^2\right) \quad , \quad \textrm{L} \in \left\{ 2,3, \ldots , \textrm{L}_{\text{ final } }\right\} \end{aligned}$$

where

8 $$\begin{aligned} i_p=\underset{\forall {\hat{i}} \ne i, l_i \ne l_{{\hat{i}}}}{\arg } \min \left\| {\varvec{x}}_{{\varvec{i}}}^L-{\varvec{x}}_{{\hat{i}}}^{\textrm{L}}\right\| \quad i_n=\underset{\forall {\hat{i}} \ne i, l_i \ne l_{{\hat{i}}}}{\arg } \min \left\| {\varvec{x}}_{{\varvec{i}}}^L-{\varvec{x}}_{{\hat{i}}}^{\textrm{L}}\right\| \end{aligned}$$

In fact, for each anchor ${\varvec{x}}_{{\varvec{i}}}^L, {\varvec{x}}_{{\varvec{i}}_p}^L$ and ${\varvec{x}}_{{\varvec{i}}_{{\varvec{n}}}}^L$ denote the positive and negative points, respectively. An interesting property of the above triplet loss is that the positive and negative points are the nearest neighbors of the anchor. Such property is consistent with the operation of a convolutional layer, which uses shared weights and local connections and hence leads to better optimization. However, in comparison to triplet loss, which uses random positive and negative data points, the above triplet loss requires computing the matrix distance of the data points.

Our proposed method is not dependent on any specific architecture and can be applied to all neural network architectures that support layer-wise addition. The training process can be facilitated using our proposed approach across various neural network structures that allow for the incremental addition of layers.

**Algorithm 1:**
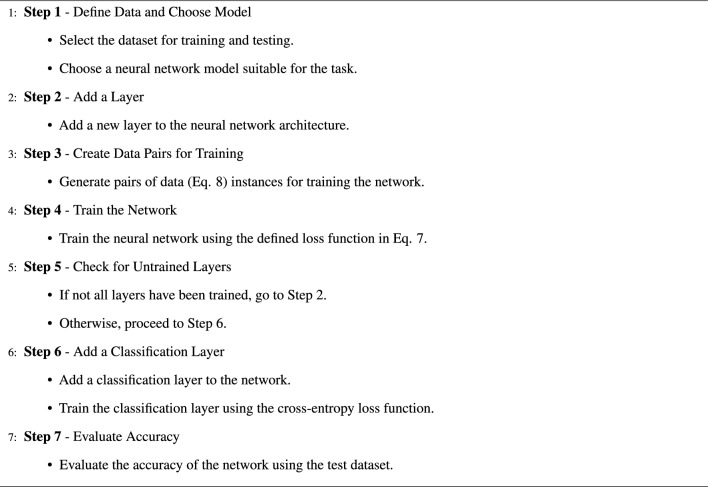
Proposed method algorithm

## Experimental results

In this section, we have conducted several experiments to show that our proposed method is more accurate than state-of-the-art methods. Our experiments have been performed in two sections: image classification and text classification. In Section “Image classification”, we will analyze the proposed method for classifying images using state-of-the-art CNNs. In Section “Text classification”, we will analyze the proposed method for classifying texts using VD-CNN^25^.

### Image classification

In this section, our proposed method is evaluated for image classification on several datasets and CNN architectures. Experiments are conducted using four architectures, namely VGG16^38^, VGG19^38^, AlexNet^39^, and LeNet^40^. The datasets utilized for training the CNNs include CIFAR-10^41^, CIFAR-100^41^, Fashion-MNIST^42^, and Raabin-WBC^43^.

#### Implementation details

The parameters used for all the image classification experiments were the same, as indicated in Table 1. Each layer was trained for ten epochs in the proposed method, while in the End-to-End method, it was trained for 200 epochs. The other hyperparameters were the same in both methods. Through parameter search and evaluation, the optimal parameters have been selected for the End-to-End learning method, and these parameters have been utilized for training using the proposed method.

Our experiments indicate that the parameter values for both methods are nearly similar, and those parameters performing well for the End-to-End method also hold suitable for our method. For instance, a learning rate of 0.5 yields unfavorable results for both methods, while a learning rate of 0.01 proves to be an appropriate value for effective learning in both approaches.

The number of epochs for the proposed method has also been determined through various experiments, representing a value that allows the network to achieve its maximum accuracy. Additionally, in End-to-End learning, a value of 200 epochs may be considered appropriate for achieving maximum accuracy throughout the learning process.

**Table 1:** Hyperparameters of neural networks used in image classification experiments.

Parameter	Value
Epoch (end-to-end)	200
Epoch per layer (proposed method)	10
Optimizer	SGD or Adam
Batch size	128
Initial learning rate	0.01

#### Datasets and architectures

In this experiment, we utilized four state-of-the-art convolutional architectures, namely VGG16, VGG19, AlexNet, and LeNet, on four benchmark datasets: CIFAR-10, CIFAR-100, Fashion-MNIST and Raabin-WBC. Table 2 provides detailed information about each dataset. The choice of these datasets was deliberate and aimed at evaluating the proposed methods across diverse domains. CIFAR-10 and CIFAR-100 are widely recognized datasets for image classification, each containing a diverse set of 60,000 32$\times$32 color images distributed over 10 and 100 classes, respectively. Fashion-MNIST is another image classification dataset consisting of 28$\times$28 grayscale images of 10 different fashion categories, providing a different context for evaluation. Additionally, Raabin-WBC, a dataset focused on medical applications, was chosen to assess the method’s performance in a domain-specific context related to medicine. Additionally, by selecting these datasets, we have evaluated the effectiveness of the proposed method in image classification across datasets with a range of categories, from a small (Raabin-WBC) to a large (CIFAR-100) number of classes.

**Table 2:** Image classification datasets used in experiments.

Dataset	Train size	Test size	Number of classes
CIFAR-10^41^	50,000	10,000	10
CIFAR-100^41^	50,000	10,000	100
Fashion-MNIST^42^	60,000	10,000	10
Raabin-WBC^43^	10,175	4339	5

#### Results

The results of the experiments indicate that our proposed method achieved higher accuracy than the End-to-End method and outperformed the layer-wise methods. Because there have been limited studies on layer-wise learning algorithms, the main comparison has been conducted between our proposed and End-to-End methods as the state-of-the-are learning methods^44^. The comparison results with the End-to-End method are presented in Table 3. Specifically, in the CIFAR-100 dataset using the VGG19 network, our proposed method achieved an accuracy of 72.49%, outperforming the End-to-End method, which achieved an accuracy of 71.23%.

Table 3 shows the performance of different learning strategies on the CIFAR-10, CIFAR-100, Fashion-MNIST and Raabin-WBC datasets using various convolutional architectures. In the CIFAR-10 dataset, our method achieved an accuracy of 93.51% for VGG16, 93.43% for VGG19, 86.25% for AlexNet, and 67.47% for LeNet. In comparison, the End-to-End method using SGD optimization achieved accuracies of 92.95%, 92.52%, 84.61%, and 67.30% for the respective architectures. When using the Adam optimization with the End-to-End method, the accuracies were slightly lower, with values of 92.86%, 92.39%, 84.50%, and 67.12%. the Forward-backward method achieved accuracies of 94.81%, 94.71%, 85.84%, and 67.61% which our method is better two case from Forward-backward learning strategy

For Fashion-MNIST, our proposed method achieved an accuracy of 95.04%, outperforming End-to-End approaches using both Cross Entropy with SGD (94.17%) and Cross Entropy with Adam (93.14%). Similarly, on the Raabin-WBC dataset, our method demonstrated superior results with an accuracy of 98.50%, surpassing the End-to-End models using Cross Entropy with SGD (97.83%) and Cross Entropy with Adam (97.94%). These findings highlight the effectiveness of our proposed method in achieving higher accuracy compared to alternative End-to-End approaches on both datasets.

**Table 3:** Comparison of accuracy between our proposed method and state-of-the-arts methods in image classification.

Dataset	Learning strategy	VGG16	VGG19	AlexNet	LeNet
CIFAR-10	Proposed method	93.51	93.43	86.25	67.47
	Forward-backward^21^	94.81	94.76	85.84	67.61
	Greedy Layer-Wise^23^	82.58	81.85	75.71	60.98
	End-to-End (Cross Entropy - SGD)	92.95	92.52	84.61	67.30
	End-to-End (Cross Entropy - Adam)	92.86	92.39	84.50	67.12
CIFAR-100	Proposed Method	72.13	72.49	62.43	53.59
	Forward-backward^21^	71.74	71.55	62.45	52.87
	Greedy Layer-Wise^23^	62.81	62.36	55.71	47.10
	End-to-End (Cross Entropy - SGD)	70.98	71.23	62.22	52.91
	End-to-End (Cross Entropy - Adam)	70.36	71.09	62.05	52.36
Fashion-MNIST	Proposed Method	95.04	95.21	92.89	73.16
	Forward-backward^21^	94.73	95.06	92.65	72.98
	Greedy Layer-Wise^23^	85.33	85.74	82.87	66.55
	End-to-End (Cross Entropy - SGD)	94.17	94.61	92.53	72.19
	End-to-End (Cross Entropy - Adam)	93.14	93.43	92.20	71.76
Raabin-WBC	Proposed method	98.50	98.35	97.56	93.31
	Forward-backward^21^	98.03	98.0	97.21	92.67
	Modified Greedy Layer-Wise^23^	93.56	92.19	91.97	88.40
	End-to-End (Cross Entropy - SGD)	97.83	97.98	96.98	92.65
	End-to-End (Cross Entropy - Adam)	97.94	98.02	96.49	92.04

As shown in Table 4, we compare our proposed method with that presented by Belilovsky^25^ et al. proposed a convolutional architecture called SimCNN, which showed promising results in previous studies. Our proposed method was compared with Belilovsky et al.’s for a comprehensive evaluation using SimCNN.

Table 4 presents a comparison of the results achieved by different learning strategies on the CIFAR-10 dataset using the SimCNN architecture. We achieved accuracies of 89.2%, 91.3%, and 93.0% for SimCNN with $k=1$, $k=2$, and $k=3$, respectively. On the other hand, the method proposed by Belilovsky et al. achieved accuracies of 88.3%, 90.4%, and 91.7% for the respective *k* values. Our results clearly indicate that we can consistently outperform Belilovsky et al.^25^ layer-wise approach on the CIFAR-10 dataset, demonstrating that our method is superior in terms of gaining higher accuracy.

**Table 4:** Comparison of accuracy between our proposed method and another layer-wise method in image classification.

Dataset	Learning strategy	SimCNN (k=1)^25^	SimCNN (k=2)^25^	SimCNN (k=3)^25^
CIFAR-10	Proposed method	89.2	91.3	93.0
	Belilovsky et al.^25^	88.3	90.4	91.7

Table 5 presents a comprehensive comparison of time complexities (in minutes and seconds) among various learning strategies, including our proposed method, Forward-backward^21^, Modified Greedy Layer-Wise^23^, End-to-End (Cross Entropy - SGD), and End-to-End (Cross Entropy - Adam), applied to the CIFAR-10 dataset. The values reflect the time each method requires to accomplish image classification tasks using different convolutional architectures (VGG16, VGG19, AlexNet, and LeNet). Our proposed method notably stands out with significantly lower time complexity, underscoring its efficiency in comparison to other state-of-the-art methods. This highlights the practicality and computational advantage of our approach in the realm of image classification.

Our proposed method is trained in fewer epochs, and the training time for the first layers is also less than the training of all layers. This is due to the fewer parameters in the network, resulting in less time for each epoch in the first layers. Only in the final layers, the time per epoch for the proposed method equals the time per epoch for the end-to-end learning method.

**Table 5:** Comparison of time complexity (minutes:seconds) between our proposed method and state-of-the-arts methods in image classification.

Dataset	Learning Strategy	VGG16	VGG19	AlexNet	LeNet
CIFAR-10	Proposed Method	102:48	129:17	52:19	16:35
	Forward-backward^21^	127:44	138:48	62:96	31:83
	Modified Greedy Layer-Wise^23^	124:49	133:26	57:56	20:42
	End-to-End (Cross Entropy - SGD)	139:43	149:58	68:28	32:38
	End-to-End (Cross Entropy - Adam)	144:57	148:45	69:12	34:20

In summary, our proposed method consistently outperformed comparable approaches in the majority of experiments, demonstrating its superior performance in various scenarios. Notably, the proposed method exhibited better predictive accuracy across different datasets and network architectures. Furthermore, in terms of computational efficiency, our method consistently surpassed its counterparts, showcasing its efficacy in achieving accurate results with reduced time complexity. These results collectively highlight the robustness and efficiency of our proposed approach, making it a promising candidate for diverse applications in comparison to similar methods.

### Text classification

In this section, we test the proposed method on two well-known data classification datasets, AG’s News and DBPedia, on the VD-CNN network^45^.

#### Implementation details

The value of the hyperparameters of the proposed method and the state-of-the-arts methods are the same as in Table 6, with the difference that the number of epochs for the End-to-End method is equal to 15, and our proposed method is trained for three epochs in each layer. The hyperparameters of the VD-CNN network have been selected based on the paper^45^, which introduces the VD-CNN network.

**Table 6:** Hyperparameters of neural networks used in text classification experiments.

Parameter	Value
Epoch (end-to-end)	15
Epoch per layer (our method)	3
Optimizer	SGD
Batch size	2048
Initial Learning Rate	0.01
Character Embedding	16

#### Datasets and architectures

We conducted experiments using the VD-CNN architecture and evaluated its performance on AG’s News and DBPedia datasets. The AG’s News dataset is designed for categorizing English news, while the DBPedia dataset is used to classify ontologies. This dataset’s details are included in Table 7.

The AG’s News dataset is specifically curated for English news categorization, encompassing various topics and subjects typically found in news articles. This dataset is well-suited for evaluating text classification models on real-world news content, providing a diverse range of topics and language usage. On the other hand, the DBPedia dataset is tailored for the classification of ontologies.

**Table 7:** Text classification datasets used in experiments.

Dataset	Train size	Test size	Number of classes
AG’s news^46^	120,000	7600	4
DBPedia^47^	560,000	70,000	14

#### Results

Based on AG’s News and DBPedia datasets, Table 8 compares the proposed and state-of-the-art methods for text classification using the VD-CNN architecture. For a comprehensive evaluation, we used three different configurations of VD-CNN with varying network depths.

The proposed method outperformed all three AG’s News dataset experimental models. The testing error rates for our method were 9.35% for VD-CNN (Depth=9), 8.71% for VD-CNN (Depth=17), and 8.72% for VD-CNN (Depth=29). On the other hand, the End-to-End method with cross-entropy loss achieved error rates of 10.17%, 9.29%, and 9.36% for the respective network depths. Also, error rates of other state-of-the-art methods are higher than our proposed method.

Similarly, in the DBPedia dataset, our proposed method demonstrated better performance compared to the End-to-End method. The testing error rates for our method were 1.53% for VD-CNN (Depth=9), 1.30% for VD-CNN (Depth=17), and 1.23% for VD-CNN (Depth=29). In contrast, the End-to-End method achieved error rates of 1.64%, 1.42%, and 1.36% for the respective network depths.

**Table 8:** Comparison of error-rate between our proposed method and state-of-the-arts methods in text classification.

Dataset	Learning strategy	VD-CNN(Depth=9)	VD-CNN(Depth=17)	VD-CNN(Depth=29)
AG’s News	Proposed method	9.35	8.71	8.72
	Forward-backward^21^	9.61	9.19	10.61
	Modified Greedy Layer-Wise^23^	13.67	12.99	11.44
	End-to-End (Cross Entropy)	10.17	9.24	9.30
DBPedia	Proposed Method	1.53	1.30	1.23
	Forward-backward^21^	1.67	1.44	1.46
	Modified Greedy Layer-Wise^23^	2.83	2.19	2.32
	End-to-End (Cross Entropy)	1.64	1.42	1.36

## Conclusion

This paper introduces a groundbreaking layer-wise learning algorithm inspired by the NGRAD hypothesis, offering a distinctive biological perspective. NGRAD posits that higher-level activities, emanating from sources such as a target, another modality, or a broader spatial or temporal context, can guide lower-level activities towards values aligned with the higher-level activity or desired output. In the context of our proposed algorithm, NGRAD serves as a guiding principle for effective synaptic updates.

Our method endeavors to train convolutional neural networks by maximizing SI. It has been discussed that maximizing SI mitigates data uncertainties and segregates data examples with different labels at each layer. In the presented algorithm, a new variant of triplet loss approximates the SI to leverage gradient-based learning methods. The proposed method undergoes evaluation in image and text classification, with its performance compared to state-of-the-art methods. According to the results, this method consistently outperforms other approaches in terms of accuracy and error rate. For instance, when applied to the CIFAR-10 dataset using the AlexNet network, our proposed method achieves an accuracy of 86.2%, surpassing the accuracy of the End-to-End learning method at 84.61%. Additionally, on the AG’s News dataset using the VD-CNN network, our proposed method achieves an error rate of 8.72%, outperforming the End-to-End method’s error rate of 9.36%.

The findings demonstrate the effectiveness and potential of the proposed method for improving the performance of deep neural networks. In future studies, our proposed method can be applied to self-supervised learning. Furthermore, the combination of layer-wise learning with SI can assist in compressing the network during training. Additionally, the applications of this approach can extend to the search for neural network architectures, facilitating the automated layer-wise design of neural networks.

## Data Availability

The datasets used and/or analysed during the current study available from the corresponding author on reasonable request.

## Code availability

The code and models can be accessed by sending a request email to alikarimi120@gmail.com.
